# Schwannoma of the Appendix Orifice

**DOI:** 10.1155/2021/7250145

**Published:** 2021-12-11

**Authors:** Maha Alkhattab, Amenah Dhannoon, Rishabh Sehgal, Conor Gormley, Margaret Sheehan, Ray Mclaughlin

**Affiliations:** ^1^Department of Surgery, Galway University Hospital, Galway, Ireland; ^2^Department of Anesthesiology, Galway University Hospital, Galway, Ireland; ^3^Department of Pathology, Galway University Hospital, Galway, Ireland

## Abstract

Schwannomas are rare mesenchymal tumors. They are usually diagnosed incidentally during endoscopic or diagnostic imaging for another reason. Malignant transformation is rare. In this case report, we present an incidental schwannoma protruding through the appendiceal orifice diagnosed during endoscopy. A healthy 56-year-old female underwent a surveillance colonoscopy for family history of colorectal cancer. A prominent and edematous appendiceal orifice was noted, and the area was aggressively biopsied. Histopathological assessment revealed a benign schwannoma. Computerized topography was unremarkable. Subsequently, the patient underwent a right hemicolectomy. Patient is scheduled to undergo routine surveillance in three years. Grossly, schwannomas are white, encapsulated, and well-circumscribed lesions that stain strongly positive for S100, GFAP, and CD57. Histologically, schwannomas demonstrate spindle cell proliferation. Several imaging modalities have been utilized in the diagnosis and management of mesenchymal neoplasms. Despite the benign nature of the diagnosis, complete surgical resection with clear margins remains the gold standard management strategy. Our case highlights the presence of a relatively uncommon tumor in an unusual anatomical location.

## 1. Introduction

Schwannomas are rare mesenchymal tumors of the gastrointestinal (GI) tract. They originate from Schwann cells of the peripheral nervous system and predominantly affect female adults with a median age of 60 [1]. Schwannomas are mostly found within the head and neck followed by limbs peripherally [2]. Rarely, they can affect the GI system with most diagnosed incidentally during routine endoscopy or imaging performed as part of another diagnostic workup [3, 4]. 3-5% of schwannomas occur in patients with neurofibromatosis type II (NF II). In the gastrointestinal tract, they are reported to occur in decreasing frequency from the stomach (83%), small intestine (12%), followed by the colon, and the rectum [4]. Despite the benign nature of the schwannomas and the extremely low risk of malignant transformation, total surgical excision remains the gold standard management option advocated for these patients [5]. Gupta et al. studied schwannomas arising from a myriad of anatomic locations in 303 patients and demonstrated that despite the benign nature of these tumors, up to 2% developed distant metastatic disease. Even though the risk of malignant transformation remains minimal, it should not be ignored [6]. We present a rare case of a schwannoma arising from within the caecal pole encroaching upon the appendiceal orifice.

## 2. Case Report

A 56-year-old female underwent an elective outpatient surveillance colonoscopy for a significant family history for bowel cancer as her sister was diagnosed with colon cancer at age 36 years. Her background medical history was significant for menorrhagia, hypertension and depression, and two caesarean sections. She had no known drug allergies, and her regular medications consisted of Nebivolol 5 mg OD, Venlafaxine 150 mg OD, and Amlodipine/Valsartan 5/180 mg.

**Figure 1:**
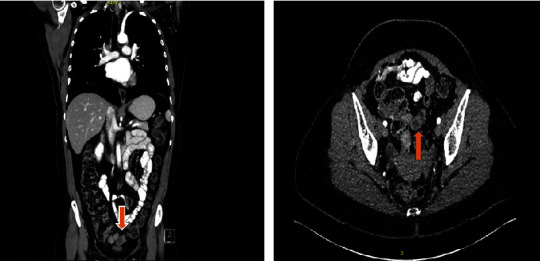
Coronal and cross-sectional views of computerized topography imaging demonstrating the appendiceal lesion.

Findings from the colonoscopy demonstrated a prominent appendiceal orifice with edematous like changes. The area was aggressively biopsied. Histopathological analysis of the biopsies demonstrated some mild neutrophilic inflammation with dense lymphocytic infiltrate of the lamina propria. The patient underwent radiologic evaluation with a computerized topography abdomen and pelvis (CTAP). Cross-sectional imaging revealed a 2.4 × 2.6 cm sized mass in the caecal tip involving the proximal appendix (Figure 1). Subsequently, she underwent a magnetic resonance imaging (MRI) which failed to further characterize the lesion. After discussing the case in the regional gastrointestinal cancer multidisciplinary team meeting in a tertiary hospital, the decision was made to proceed with a right hemicolectomy.

**Figure 2:**
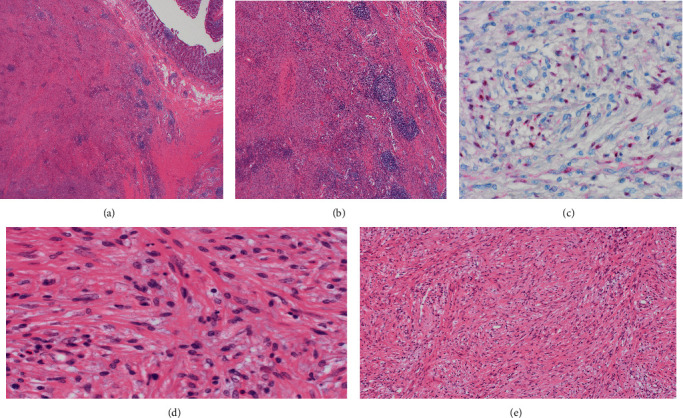
(a) Spindle cell lesion in appendix wall predominantly in muscularis propria. (b) Associated benign lymphoid reaction-typical of gastrointestinal GI schwannoma. (c) S100 positivity in the lesion. (d) Bundles of bland spindle cells (high power). (e) Bundles of bland spindle cells (low power).

The index patient underwent a right hemicolectomy in November 2019. Gross pathology of the surgical specimen demonstrated a cream solid cut surface homogenous throughout. It appeared to be arising from the proximal base of the appendix/appendiceal orifice. Microscopic evaluation of the lesion demonstrated a 22 mm encapsulated well-circumscribed spindle cell mesenchymal neoplasm arising in the cecum with low-grade morphology. Due to the rare and unusual histology and location, the specimen was referred the Brigham and Women's Hospital Boston/Harvard Medical School for a second expert opinion. The additional histopathology was reported as a well-circumscribed submucosal lesion composed mainly of bland spindle cells. There was no atypia. Immunohistochemical stains showed diffuse positivity for S100 protein and SOX10 while CD34 and EMA were negative. The findings were in keeping with a benign schwannoma (Figure 2). Postoperative recovery was uneventful and was discharged well on postoperative day four. On the 6-week outpatient review, the patient was completely asymptomatic. Patient is scheduled to undergo routine surveillance in three years.

## 3. Discussion

Schwannomas are mesenchymal tumors arising from the Schwann cells. They account for approximately 5% of mesenchymal neoplasms [7]. The stomach is the most frequently encountered site of schwannomas along the gastrointestinal tract, yet it contributes to 0.2% of all diagnosed gastric neoplasms [8]. However, it is much less common than gastrointestinal stromal tumors (GISTs) at this site [1].

Differential diagnosis includes gastrointestinal stromal tumors, leiomyomas, and solitary neuroendocrine (carcinoid) tumors [9]. Occasionally, lymphomas and GI adenocarcinomas may demonstrate an intramural growth and could mimic mesenchymal tumors [9].

It has been established that schwannomas can vary in size from few millimeters to greater than 20 cm in diameter. The small lesions' morphology has been described as white, fusiform, circumscribed, and encapsulated similar to that described in the current case presented, as opposed to the larger ones that would mainly be lobulated and irregular in appearance [6]. On histological assessment, these tumors are typically composed of spindle cells with variable myxoid stroma. Immunohistochemically, schwannomas stain strongly positively for S100 and focally for GFAP and CD57 [10]. Generally, these morphological, histological, and immunohistochemical findings in the absence of KIT positivity and smooth muscle markers are sufficient to confirm the diagnosis [1]. GIST is the main differential diagnosis to be ruled out when schwannoma is suspected. These two tumors are similar histologically in that they both demonstrate a spindle-like proliferation. They however are immunohistochemically distinct. Greater than 95% GISTs express c-Kit (CD117), CD34 (70%), and H-caldesmon (80%) [11].

Various imaging modalities were used to distinguish schwannomas from other gastric mesenchymal neoplasms including endoscopic ultrasound, computerized topography (CT), and magnetic resonance imaging (MRI) in addition to a positron emission topography (PET) scan [3]. Wang et al. investigated the ultrasonic features of 4 gastric schwannomas noted during endoscopic ultrasound evaluation. The internal echogenicity of these tumors was heterogeneous and low, but slightly higher than that of muscularis propria [12]. Endoscopic ultrasound is proven to be of a good diagnostic value when the lesion in question is located within the stomach [4]. Schwannomas appear as well-defined transmural hypoechoic masses on ultrasound imaging [4]. Levy et al. [13] investigated the radiological features of this rare mesenchymal neoplasm in 8 cases of histopathologically proven schwannomas retrospectively. The authors concluded that schwannomas generally appear as well-defined homogeneously attenuating mural masses on CT. They lack the poor prognostic factors seen typically in gastrointestinal stromal tumors such as low attenuating hemorrhage, necrosis, or degradation within the tumor [13]. MRI findings of GI schwannomas suggest they follow the same pattern of those diagnosed in the head and neck region [14]. They appear as low to isotense lesions in T1-weighted images while isotense to high tense on T2-weighted images [14]. Ohno et al. reported two cases of gastric schwannomas that were investigated with PET CT scan. This revealed an increased [18F] fluorodeoxyglucose (FDG) uptake on positron emission tomography [8]. This case report highlights schwannoma as an important differential diagnosis in suspected tumors in the gastrointestinal tract. Various cases have been reported in literature. Bajaj et al. [15] published clinical guidance on the diagnosis and management of mesenchymal neoplasms in Science direct in April 2020. It is recommended that patients with underlying confirmed gastrointestinal schwannomas undergo complete surgical excision. Partial excision is also possible if the tumor is large and there is a risk of nerve damage. It has been reported that even with partial excision, malignant transformation remains extremely rare [16]. However clinical guidance is still significantly lacking.

## 4. Conclusion

Schwannoma is a rare mesenchymal neoplasm that has the tendency to affect various organs including the gastrointestinal tract. They are benign tumors with low malignant potential. Our case highlights the significance of having a broad range differential diagnosis when a lesion is identified endoscopically or during cross-sectional imaging.
